# *Dlx5/6* Expression Levels in Mouse GABAergic Neurons Regulate Adult Parvalbumin Neuronal Density and Anxiety/Compulsive Behaviours

**DOI:** 10.3390/cells11111739

**Published:** 2022-05-25

**Authors:** Rym Aouci, Mey El Soudany, Zakaria Maakoul, Anastasia Fontaine, Hiroki Kurihara, Giovanni Levi, Nicolas Narboux-Nême

**Affiliations:** 1 Physiologie Moléculaire et Adaptation, CNRS UMR7221, Team BBC, Département AVIV, Muséum National d’Histoire Naturelle, UMR-7221, 7 rue Cuvier, 75005 Paris, France; rym.aouci@mnhn.fr (R.A.); mey_labsse75019@hotmail.fr (M.E.S.); maakoul.zakaria.k@gmail.com (Z.M.); anastasia.fontaine@mnhn.fr (A.F.); glevi@mnhn.fr (G.L.); 2 Department of Physiological Chemistry and Metabolism, Graduate School of Medicine, The University of Tokyo, Tokyo 113-0033, Japan; kuri-tky@umin.net

**Keywords:** GABAergic neurons, *DLX5/6* genes, parvalbumin, behaviour, gene regulation, autism, schizophrenia, hippocampus, prelimbic cortex

## Abstract

Neuronal circuits integrating Parvalbumin-positive GABAergic inhibitory interneurons (PV) are essential for normal brain function and are often altered in psychiatric conditions. During development, *Dlx5* and *Dlx6* (*Dlx5/6*) genes are involved in the differentiation of PV-interneurons. In the adult, *Dlx5/6* continue to be expressed at low levels in most telencephalic GABAergic neurons, but their importance in determining the number and distribution of adult PV-interneurons is unknown. Previously, we have shown that targeted deletion of *Dlx5/6* in mouse GABAergic neurons (*Dlx5/6^VgatCre^* mice) results in altered behavioural and metabolic profiles. Here we evaluate the consequences of targeted *Dlx5/6* gene dosage alterations in adult GABAergic neurons. We compare the effects on normal brain of homozygous and heterozygous (*Dlx5/6^VgatCre^* and *Dlx5/6^VgatCre/+^* mice) *Dlx5/6* deletions to those of *Dlx5* targeted overexpression (*GABAergic^Dlx5/+^* mice). We find a linear correlation between *Dlx5/6* allelic dosage and the density of PV-positive neurons in the adult prelimbic cortex and in the hippocampus. In parallel, we observe that *Dlx5/6* expression levels in GABAergic neurons are also linearly associated with the intensity of anxiety and compulsivity-like behaviours. Our findings reinforce the notion that regulation of *Dlx5/6* expression is involved in individual cognitive variability and, possibly, in the genesis of certain neuropsychiatric conditions.

## 1. Introduction

Brain function depends on neuronal microcircuits composed of excitatory neurons responsible for long- and short-range signal transmission and regulatory GABAergic inhibitory interneurons. The tuning of these neuronal networks affects most brain functions, including cognition, perception [1] and social behaviour [2]. GABAergic interneurons constitute a heterogeneous class of cells which can be classified on the basis of their peculiar anatomical, biochemical and physiological features [3]. They were divided into three major classes characterised by the expression of either parvalbumin (PV), somatostatin (SST) or serotonin receptor 3A (5HTr3A), although the great diversity of GABAergic interneurons is only now appreciated due to single cell transcriptomic analysis and has resulted in the identification of more than 20 sub-classes [4,5]. These diverse adult GABAergic subtypes are generated through well-defined transcriptional trajectories in which the sequential expression of groups of transcription factors (TFs) leads to the progressive differentiation of neuronal progenitors [6]. Subtle variations in GABAergic differentiation could be at the origin of individual cognitive differences, but also of neuropsychiatric conditions. For example, reduced firing of PV-interneurons, which leads to an increased excitation to inhibition ratio (E/I), could be one of the underlying causes of ASD [7,8,9].

*Dlx* genes constitute a family of transcription factors sharing a conserved homeodomain; they are involved in several developmental processes [10,11], including neuronogenesis [12]. In mammals, six *Dlx* genes are arranged in three bigenic clusters: *Dlx1/Dlx2*, *Dlx3/Dlx4* and *Dlx5/Dlx6* [13]. *Dlx5* and *Dlx6* are expressed by developing and mature GABAergic interneurons [10,14,15,16,17,18] and are particularly important for the differentiation of PV-interneurons. Indeed, when immature *Dlx5/6*-null interneurons are transplanted into wild-type newborn brains, most fail to differentiate into PV-positive GABAergic neurons, although other GABAergic subtypes are unaffected [19]. Mice carrying a heterozygous *Dlx5/6* systemic inactivation show defects in prefrontal cortex gamma (γ; ∼30–120 Hz) oscillations, which depend on PV-interneurons activity, resulting in working memory deficits [20].

We have recently shown that targeted inactivation of *Dlx5* and *Dlx6* in mouse GABAergic interneurons affect behaviour, vocal socialisation and metabolism with a reduction in anxiety-like and obsessive-compulsive-like behaviours [15,21].

*Dlx5* gives rise to two major mRNA transcripts. The larger transcript, which includes the DNA-binding domain, is mostly found in the nucleus and acts as a transcriptional regulator; the shorter transcript lacks the homeodomain. The long and short forms of the DLX5 protein coexist in normal neurons and are both capable of participating in protein complexes with other partners, including MAGED1 and NECDIN during GABAergic differentiation [22,23]. The relative contribution of the two DLX5 isoforms to neuronal differentiation and function is still unknown.

Recently, a large-scale transcriptomic study on post-mortem brains has suggested that the *DLX5/6* locus participates in genetic modules altered in ASDs and schizophrenia [24].

In the human genome, *DLX5* is located on chromosome 7q21.3 in a region imprinted in lymphoblasts and brain tissues [25] but is biallelically expressed in the mouse with preferential transcription of the maternal allele. This genomic region has been shown to be a target of MECP2 and to be deregulated in Rett syndrome, an X-linked neurodevelopmental disorder [26].

Deletions or mutations at the *DLX5/6* locus are associated with split hand foot malformation type 1 (SHFM1), an ectrodactyly often associated with cognitive, craniofacial or hearing defects [21,27]. Large heterozygous deletions covering both regulatory and coding *DLX5/6* sequences present with SHFM1 and craniofacial defects often associated with mental retardation. Patients carrying mutations in the *DLX5/6* intergenic region, including three brain-specific enhancers *I56i, I56ii* and *MEF2* [28,29], present a higher incidence of autism spectrum disorder (ASD) or speech delay without limb deformities [30,31,32,33].

Loss of the *I56i* intergenic enhancer alters the level of expression of *Dlx5/6* and results in a decrease in GABAergic cells in the developing forebrain; these mutants demonstrate increased sociability and learning deficits [28,34]. Mutations involving brain *Dlx5/6* regulatory regions are, therefore, associated with cognitive abnormalities both in humans and mice.

The aim of this study is to better understand how the level of *Dlx5/6* expression in GABAergic interneurons affects the brain’s cellular composition and function. To this end, we compare mice in which the DNA-binding regions of *Dlx5* and *Dlx6* have been deleted in GABAergic neurons either in one or both alleles (*Dlx5/6^VgatCre/+^* and *Dlx5/6^VgatCre^* mice, respectively) to mice in which the overexpression of *Dlx5* is forced in all GABAergic neurons using a conditional knock-in of *Dlx5* in the ROSA locus (*GABAergic^Dlx5/+^* mice) [35]. We show that increasing or decreasing *Dlx5/6* expression in GABAergic neurons results in opposite cellular and behavioural phenotypes.

The level of *Dlx5/6* expression seems, therefore, to be essential for maintaining the equilibrium between the populations of inhibitory PV interneurons and that of excitatory neurons. DLX5/6 could therefore play a role in determining the excitatory/inhibitory (E/I) balance, a parameter which has been associated to neuropsychiatric conditions such as ASD and schizophrenia [36,37].

## 2. Methods

### 2.1. Animals

Mice were housed in light, temperature (21 °C) and humidity (50–60% relative humidity) controlled conditions. Food and water were available ad libitum. Mice were individually identified by a microchip postnatally implanted 3 weeks. Litter sizes and genotypes were recorded. WT animals were from Charles River, France. *Slc32a1^tm2(cre)Lowl^* knock-in mice (here referred as *Vgat^cre/+^* mice) were purchased from Jackson Laboratories through Charles River, France. In the developing telencephalon, Vgat was expressed in post-mitotic GABAergic neurons staring at E11.5 [38]. All mutant strains were backcrossed and bred on a mixed C57BL6/N X DBA/2N genetic background.

**Figure 1:**
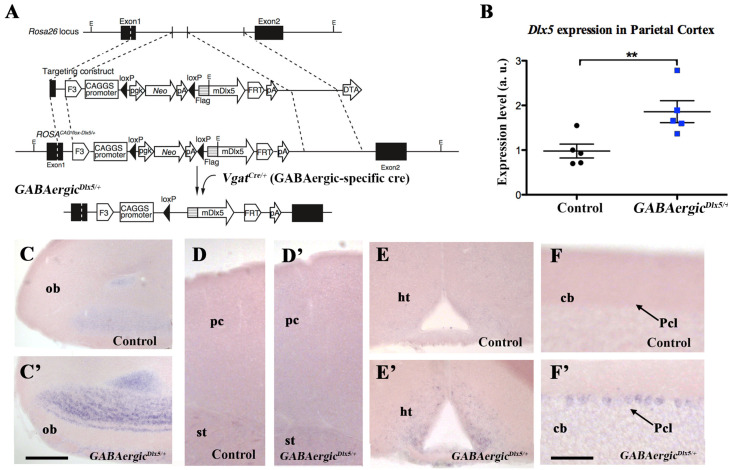
***Dlx5* expression in brain areas of *GABAergic^Dlx5/+^* adult animals** (**A**) *GABAergic^Dlx5/+^* animals were generated crossing *R26R^CAG-flox-Dlx5/+^* [35] and *Vgat^cre/+^* mice to induce GABAergic-specific expression of *Dlx5* and then backcrossed for more than 10 generations on a mixed C57BL6/N X DBA/2N genetic background. (**B**) qPCR analysis of *Dlx5* expression level in the parietal cortex of control and *GABAergic^Dlx5/+^* littermates. The level of *Dlx5* expression almost doubled in animals compared with controls. (**C**–**F’**) Dlx5 expression was revealed by in situ hybridisation performed in parallel and with identical conditions on serial sections of young adult brains from control (**C**–**F**) and *GABAergic^Dlx5/+^* (**C’**–**F’**) mice. Strong induction of *Dlx5* expression was observed in all GABAergic areas, including the olfactory bulb (**C**,**C’**), the parietal cortex (**D**,**D’**), the hypothalamus (**E**,**E’**) and the cerebellum (**F**,**F’**). *Dlx5* expression was also detected in GABAergic neurons not normally expressing the gene, such as, for example, Purkinje cells in the cerebellum (**F**,**F’**). cb, cerebellum; ht, hypothalamus; ob, olfactory bulb; pc, parietal cortex; Pcl, Purkinje cells layer. Bar: 250 μm C–E’; 100 μm F–F’. Levels of significance (** *p* ≤ 0.01).

To obtain mice carrying the *R26R^CAG-flox-Dlx5/+^* allele, an *F3*/*FRT*-flanked cassette containing the CAG promoter, a floxed stop sequence, flag-tagged mouse *Dlx5* cDNA and a poly(A) additional signal were inserted into the targeting vector pROSA26-1 (P. Soriano, Mount Sinai School of Medicine, New York, NY, USA) (Addgene, plasmid 21714) [35]. *R26R^CAG-flox-Dlx5/+^* mice were crossed with *Vgat^cre/+^* to induce GABAergic-specific expression of *Dlx5* (*GABAergic^Dlx5/+^* mice) and then backcrossed for more than 10 generations on a mixed C57BL6/N X DBA/2N genetic background (Figure 1A).

*Dlx5/6^VgatCre/+^* and *Dlx5/6^VgatCre^* mice were obtained as previously described by breeding a *Dlx5/6^flox/flox^* strain with *Vgat^cre/+^* mice [21].

Mice of both sexes were used. In all experiments, littermates with no *Vgat^cre^* alleles were used as controls. Results obtained with controls from *GABAergic^Dlx5/+^* and *Dlx5/6^VgatCre^* mice for either cortical cell counting or behavioural tests were compared and no significant differences were observed.

### 2.2. Behavioural Tests

Behavioural procedures were conducted between 9 a.m. and 5 p.m. in a dim and quiet room, not housing any other animal. Observers were blind to the experimental design. Mice were taken to the test room 30 min before the test and left in the absence of the observer.

− *Open Field Test with object exploration*

We used an open field test (OFT) with a centrally located object to measure anxiety-like and exploratory behaviours of mice placed in a novel environment [39]. The equipment consisted of a closed square arena (72 × 72 cm). The computer defined the grid lines dividing the box floor into 16 equal-sized squares, with the central four squares regarded as the central region. First, mice were familiarised with the empty arena for 10 min and placed back in their home cage for 2 min. A cylindrical plastic tube (diameter 3 cm, height 7 cm) (object, OB) was placed in the centre of the arena. Each mouse was then placed at one corner of the arena facing the wall and tracked and recorded for 10 min. Films were analysed by Ethovision system (Noldus). Latency to the first entry in the centre, number of entries in the centre, latency to interact with the OB, number of interactions with the OB and duration of OB sniffing were analysed. To eliminate olfactory cues, the equipment was thoroughly cleaned between each test.

− *Marble burying test (MBT)*

We used the marble-burying test (MBT) to measure anxiety- and compulsive-like behaviours. A clear Plexiglas box (36.5 cm long × 20.7 cm wide × 14 cm high) was filled with 3 cm of standard bedding. A total of 20 glass marbles were placed on the surface of the shavings. Mice were individually placed in the centre of the box and left for 10 min. At the end of the session, a picture of the marbles was taken, and the marbles buried index was counted with the Fiji (ImageJ) image-processing program.

− *Nest building test*

Each mouse, aged less than one year, was housed in a single cage before testing. During the test, a paper towel (30 cm × 21,5 cm) was placed in the cage and left for one week. The nest quality was scored daily at 10 a.m. into four categories as shown in [15]: 1-no interaction with intact paper, 2-paper partially torn, 3-paper completely torn, and 4-nest completely built.

### 2.3. Immunohistochemistry

Animals were deeply anesthetised and perfused intracardially with 4% paraformaldehyde in phosphate buffer. Brains were removed and postfixed overnight at 4 °C in the same fixative and cryoprotected by immersion in 30% sucrose. Cryoprotected brains were embedded in OCT and 60-micron thick free-floating cryostat sections were prepared. Immunohistochemical detection of parvalbumin (PV) was performed on these floating cryosections. All sections were washed twice with PBS and then treated in PBS Triton 0.1% H_2_O_2_ overnight at 4 °C. Sections were pre-treated in PBS Triton 0.1% H_2_O_2_ overnight at 4 °C, blocked in PBS 1×, 2% gelatine and 0.25% Triton and incubated with 1:1000 mouse anti-PV primary antibody (P3088, Sigma, France) overnight at 4 °C. Sections were incubated for 2 h in peroxidase-coupled goat anti-mouse antibody (1:300 Vector Laboratories, France) and revealed with 3,3′-diaminobenzidine. Sections were collected on Super Frost Ultra Plus slides (Thermo Fisher Scientific, Illkirch-Graffenstaden, France), dehydrated and mounted in Eukitt^®®^ mounting medium (03989, Sigma, St. Quentin Fallavier, France).

### 2.4. In Situ Hybridisation

In situ hybridisation was performed on 60 µm-thick free-floating frozen sections as previously described [40] with minor modifications. Then, 5-bromo-4-chloro-3-indolyl phosphate (BCIP)/nitro blue tetrazolium (NBT) was used as the revelation substrate of alkaline phosphatase.

### 2.5. Quantification of PV Neuronal Density

After immunochemistry, brain sections were photographed and specific cortical areas were delimited. PV-neurons were counted manually with the “Fiji” program counting tool and their density was calculated by dividing this number by the surface area for the frontal and somatosensory cortex. Neuronal counting in the hippocampus was normalised with the length of the CA or DG. This operation was repeated on the left and right hemi-cortices and the results were averaged.

### 2.6. Reverse Transcription Quantitative PCR (RT-qPCR)

Cortical fragments were microdissected under a stereomicroscope of 1mm thick sections in cold phosphate buffer and immediately frozen in dry ice. Total RNA was isolated from fragments dissected from control, *GABAergic^Dlx5/+^*, *Dlx5/6^VgatCre/+^* and *Dlx5/6^VgatCre^* mice using a RNeasy minikit (Qiagen) according to the manufacturer instructions. On-column deoxyribonuclease digestion (Qiagen) was applied after the RNA isolation procedure to remove potential genomic DNA contamination. cDNA was synthesised from 1 μg of RNA (Invitrogen). Real-time PCR was performed using the SYBR Green method according to the manufacturer’s instructions (SYBR Green I master, Light cycler 480, Roche Diagnostics). The comparative Ct method on MxPro qPCR software (Agilent Technologies) was used to determine the normalised changes of the target gene relative to a calibrator reference. mRNA quantification samples were normalised to peptidylprolyl isomerase A (PPIA), hypoxanthine phosphoribosyltransferase (HPRT) and phosphoglycerate kinase 1 (PGK1) levels. As a calibrator reference, we referred to Ct from RNAse-free treated water animal samples.

*Dlx5* transcripts were analysed using the following primers (Table 1):

### 2.7. Statistical Analysis

Unpaired *t*-tests, linear regression and ANOVA were performed using Prism (Graphpad Software, La Jolla, CA, USA) followed by Tukey’s multiple comparisons test. All values were expressed as means ± SEM of combined data from replicate experiments; levels of significance (* *p* ≤ 0.05, ** *p* ≤ 0.01, *** *p* ≤ 0.001, **** *p* ≤ 0.0001).

## 3. Results

### 3.1. Deregulation of Dlx5/6 Expression in Mouse GABAergic Neurons

To generate *GABAergic^Dlx5/+^* mice, in which the expression of *Dlx5* is ectopically induced in all GABAergic neurons, we crossed *ROSA^CAG-flox-Dlx5/+^* mice, which express *Dlx5* in a Cre-dependent manner [35], with *Vgat^cre/+^* mice in which an IRES-Cre recombinase cassette is inserted downstream of the stop codon of the endogenous *Vgat* (vesicular GABA transporter) gene (Figure 1A). In *Vgat-cre* mice, *Cre-recombinase* expression is observed in all GABAergic neurons but not in other cell types [41]. qRT-PCR analysis showed that the level of *Dlx5* expression in the adult parietal cortex of *GABAergic^Dlx5/+^* mice was almost doubled when compared with control littermates (Figure 1B). In situ hybridisation of serial brain sections demonstrated an increased expression of *Dlx5* in telencephalic regions known to present an endogenous level of expression in the adult (see also [15], Figure 1), such as the olfactory bulb (Figure 1C,C’), the parietal cortex (Figure 1D,D’) and the hypothalamus (Figure 1E,E’). Remarkably, in *GABAergic^Dlx5/+^* mice, *Dlx5* expression was also observed in non-telencephalic GABAergic neurons where the gene is not normally expressed during development and in the adult such as, for example, Purkinje cells of the cerebellum (Figure 1F,F’). *GABAergic^Dlx5/+^* mice did not present any obvious anatomical, metabolic or motility problems.

*Dlx5* and *Dlx6* were both inactivated in GABAergic interneurons since these two closely associated genes present similar regulations and redundant functions [16,17]. To this end, *Dlx5/6^flox/flox^* mice, in which the homeodomain-encoding regions of both *Dlx5* and *Dlx6* are flanked by non-compatible *lox* sequences [42] were crossed with *Vgat^cre/+^* mice as previously described [15]. RT-PCR analysis has shown that *Dlx5* exon II and *Dlx6* transcripts are strongly reduced in the cortex of heterozygous *Dlx5/6^VgatCre/+^* mice and virtually absent in homozygous *Dlx5/6^VgatCre^* mice [15].

### 3.2. Effects of Dlx5/6 Deregulation in GABAergic Neurons on Adult PV-Interneurons Density

Dlx5/6 are known to play a role in the development of PV-interneurons, however their effects on this population of cells in the adult are still partially understood.

Serial sections of control, *GABAergic^Dlx5/+^*, *Dlx5/6^VgatCre/+^* and *Dlx5/6^VgatCre^* adult mouse brains were immuno-stained with anti-PV antibodies and PV-positive cells were counted in defined brain regions. No significant difference in brain morphology of the cortical layer thickness was observed. Analysis of a primary cortical region and the parietal cortex, did not show any significant variation in PV-interneurons density even when each individual cortical layer was counted. As we had previously described an inhibition of threat response and reduction in anxiety-like and obsessive-compulsive activities in *Dlx5/6^VgatCre^* adult mice, we focused on the prelimbic cortex and on the hippocampus, two brain regions known to interact in the process of fear inhibition [43].

**Figure 2:**
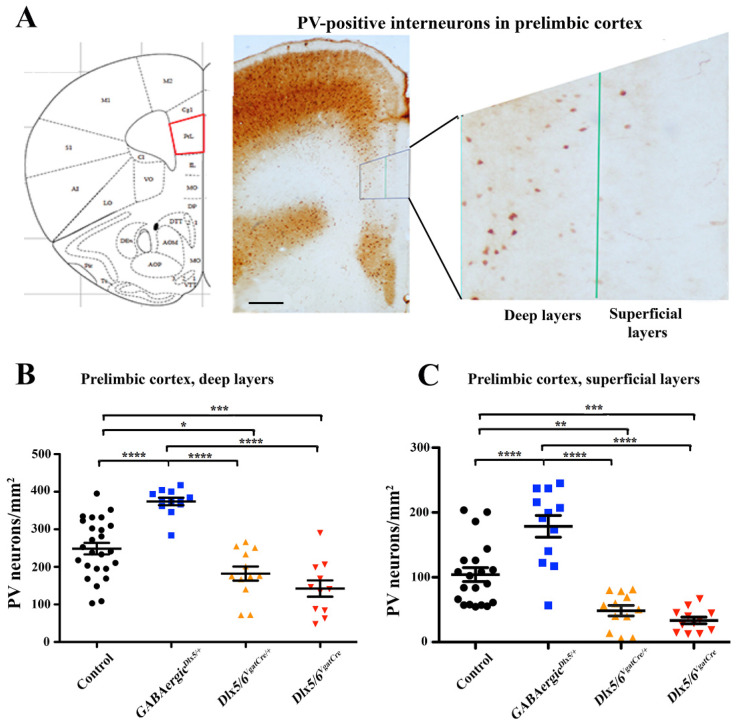
**PV neuronal density in the prelimbic cortex** (**A**) Serial sections of 6-month-old mice of different genotypes were stained with anti-PV antibodies and the density of PV-positive neurons was measured in the deep and superficial layers of the prelimbic cortex. Bar: 500 µm in the central panel; 100 μm in the enlargement. (**B**,**C**) Distribution of individual neuronal densities in the prelimbic cortex from control (n = 25), *GABAergic^Dlx5/+^* (n = 12), *Dlx5/6^VgatCre/+^* (n = 12) and *Dlx5/6^VgatCre^* (n = 11) mice. Levels of significance (* *p* ≤ 0.05, ** *p* ≤ 0.01, *** *p* ≤ 0.001, **** *p* ≤ 0.0001).

**Figure 3:**
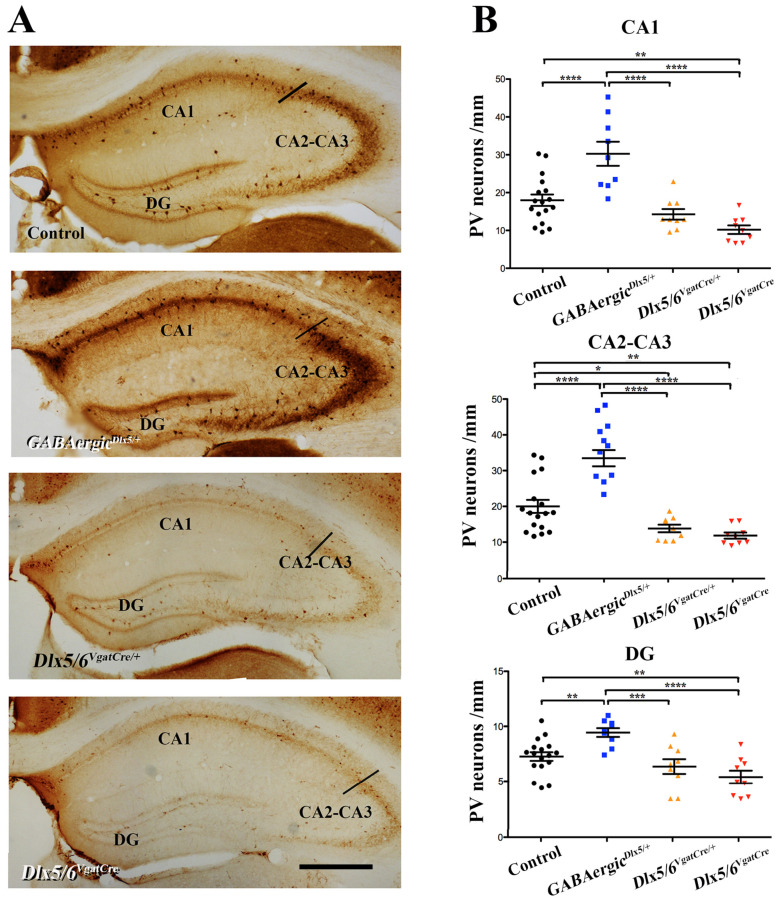
**PV neuronal density in different regions of the hippocampus** (**A**) Serial sections of 6-month-old mice of different genotypes were stained with anti-PV antibodies and the density of PV-positive neurons was measured in the three major hippocampal regions, namely CA1, CA2-CA3 and DG. (**B**) Distribution of individual neuronal densities in hippocampal regions of control (n = 9), *GABAergic^Dlx5/+^* (n = 9), *Dlx5/6^VgatCre/+^* (n = 9) and *Dlx5/6^VgatCre^* (n = 9) mice. Bar: 500 μm. Levels of significance (* *p* ≤ 0.05, ** *p* ≤ 0.01, *** *p* ≤ 0.001, **** *p* ≤ 0.0001).

**Figure 4:**
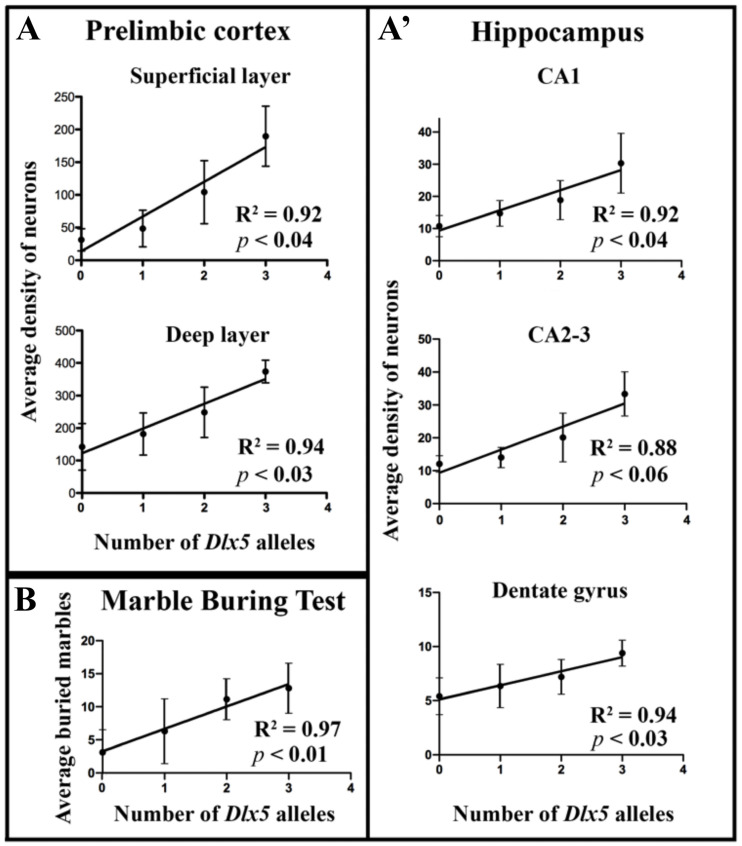
**Correlation of PV neuronal density and MBT score with *Dlx5* alleles number** (**A**,**A’**) The densities of PV-positive neurons in the superficial and deep layer of the prelimbic cortex (**A**) and in three regions of the hippocampus (**A’**) (see Figure 2 and Figure 3) were plotted in function of the number of *Dlx5* alleles present in the different genotypes namely no allele in *Dlx5/6^VgatCre^*, one allele in *Dlx5/6^VgatCre/+^,* two alleles in control, and three alleles in *GABAergic^Dlx5/+^* mice. The coefficient of determination R^2^ was calculated to evaluate the fitting of a linear regression model. (**B**) A similar analysis was performed by plotting the number of buried marbles in the MBT performed by mice of different genotypes (Figure 6). With the exception of the CA2-3 region of the hippocampus, all the R2 values were higher than 0.9 and the *p*-values were lower than 0.05 suggesting a good fitting of linear correlation models between the number of *Dlx5* alleles expressed in GABAergic neurons and both PV neuronal density and obsessive behaviours.

In both the deep (DLPL) and superficial (SLPL) layers of the prelimbic cortex (Figure 2) and in all regions of the hippocampus (namely CA1, CA2-CA3 and DG) (Figure 3) we observed a strong increase in PV neuronal density in *GABAergic^Dlx5/+^* and a reduction in PV neuronal density in *Dlx5/6^VgatCre/+^* and *Dlx5/6^VgatCre^* adult mouse brains. On average, in *GABAergic^Dlx5/+^* PV neuronal density increased by 34%, 42%, 39%, 40% and 23%, in DLPL, SLPL, CA1, CA2-CA3 and DG, respectively; in *Dlx5/6^VgatCre/+^* mice PV density diminished by 51%, 73%, 51%, 58% and 33% in DLPL, SLPL, CA1, CA2-CA3 and DG, respectively and, in *Dlx5/6^VgatCre^* mice PV density diminished by 62%, 81%, 65%, 64% and 43%, in DLPL, SLPL, CA1, CA2-CA3 and DG, respectively. In all analysed regions, we observed a significant linear correlation between the density of PV interneurons and the number of *Dlx5* alleles present in GABAergic neurons (Figure 4). It should be noted, however, that in *Dlx5/6^VgatCre^* mice, in the absence of *Dlx5/6* expression, between 20% (SLPL) and 57% (DG) of PV-interneurons continued to be present in all regions. In the parietal cortex, most PV-interneurons were still present independent of the genotype.

### 3.3. Behavioural Consequences of Dlx5/6 Expression Deregulation in GABAergic Neurons

We analysed the effects of modulating *Dlx5/6* expression in GABAergic interneurons in three experimental settings related to anxiety-like and obsessive-compulsive behaviours.

− Open Field Test with object exploration

Novel stimuli, such as unfamiliar environments or objects, are known to create conflict in rodents, concomitantly evoking exploratory and avoidance behaviours [44,45]. The latter is often interpreted as “anxiety-like” behaviours and reflect the animal’s fear of novelty.

**Figure 5:**
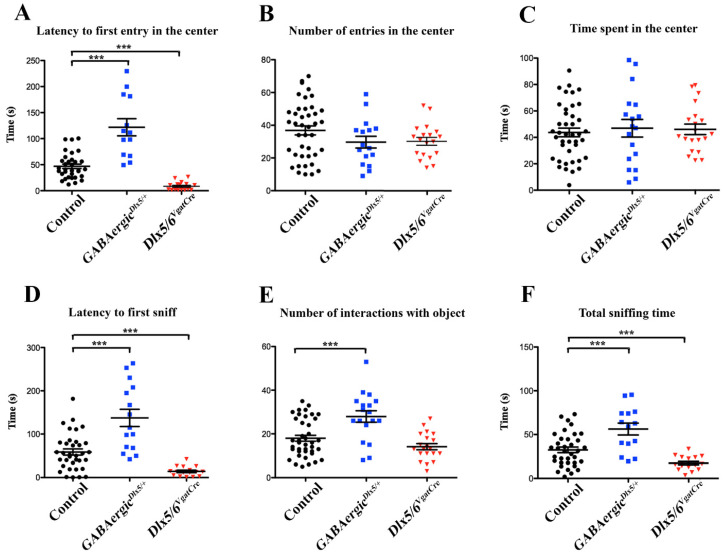
**Open Field Test with object exploration** (**A**–**F**) Different parameters associated with the propensity to enter the centre (**A**–**C**) or to interact with the object (**D**–**F**) were measured in mice of different genotypes performing in the Open Field Test with object exploration. Control (n = 33), *GABAergic^Dlx5/+^* (n = 13) and *Dlx5/6^VgatCre^* (n = 17). Levels of significance *** *p* ≤ 0.001).

In a previous paper, we have analysed the response of control, *Dlx5/6^VgatCre^* and *Dlx5/6^VgatCre/+^* mice placed in a 72 × 72 cm square flat arena for 10 min (Open Field Test, OFT), showing that the reduction in *Dlx5/6* expression in GABAergic neurons is associated with a significant decrease in anxiety-like behaviours as both homozygous and heterozygous mutant mice entered the arena more promptly and spent more time in the centre than control animals (on average 50 s vs. 100 s latency to enter the centre) ([15] Figure 4). In this study, after a 10 min period of familiarisation, the mice were reintroduced in the same arena, but in the presence of a novel object placed in the centre [46]. This experimental paradigm stimulates a higher approach and exploratory behaviours compared to the OFT. By comparing different responses of mice exposed to the OFT and to the “OFT with object exploration” it is possible, in principle, to discern exploratory from anxiety-like behaviours [44]. Indeed, in the “OFT with object exploration” it took about 50 sec for control mice to reach the object in the centre of the arena (Figure 5A) whereas a similar control group took an average of 100 s to enter the centre in the absence of an object [15]. Remarkably this enhanced exploratory behaviour, induced by the object, was exacerbated in *Dlx5/6^VgatCre^* mice, and on average, entered the central area in less than 10 s (Figure 5A) compared to the about 50 s in the absence of the object [15]. On the contrary, *GABAergic^Dlx5/+^* mice hesitated on average more than two minutes before entering the centre (Figure 5A) and did not display any different behaviour in the presence or in the absence of the object, suggesting a high level of anxiety which prevailed over exploratory inputs. However, after entering the centre the first time, within 10 min of the test, the number of entries and the time spent in the centre were not significantly different between genotypes (Figure 5B,C). Overexpression of *Dlx5* was associated with a delay in first contact with the object, but with a prolonged time of examination whereas *Dlx5/6^VgatCre^* mice went almost immediately to sniff the object but rapidly lost interest (Figure 5D–F).

− Marble burying test (MBT)

**Figure 6:**
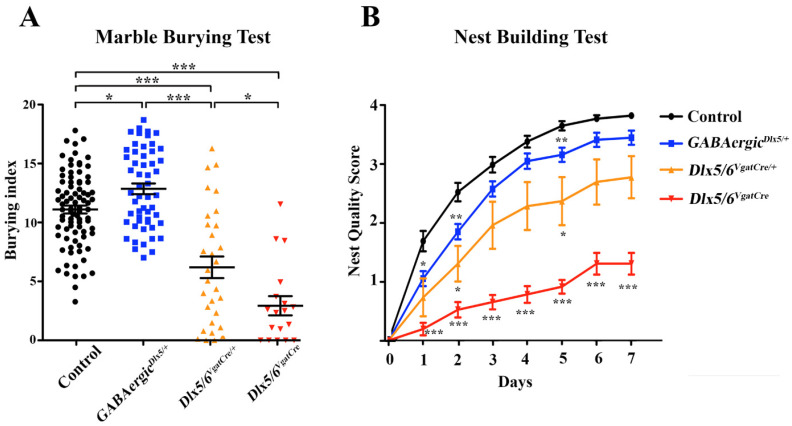
**MBT and nest building behaviours** (**A**) Burying index of mice of different genotypes performing the marble burying test. Controls (n = 85), *GABAergic^Dlx5/+^* (n = 54), *Dlx5/6^VgatCre/+^* (n = 30) and *Dlx5/6^VgatCre^* (n = 18) mice. (**B**) Nest quality scores measured in the nest building test in mice of different genotypes. Control (n = 40), *GABAergic^Dlx5/+^* (n = 27), *Dlx5/6^VgatCre/+^* (n = 15) and *Dlx5/6^VgatCre^* (n = 12) mice. Levels of significance (* *p* ≤ 0.05, ** *p* ≤ 0.01, *** *p* ≤ 0.001).

The consequences of *Dlx5/6* inactivation in GABAergic neurons on stereotyped repetitive behaviour were assessed through the Marble Burying Test (MBT) (Figure 6A).

Similarly to our previous report, during the 10 min test, both *Dlx5/6^VgatCre/+^* and *Dlx5/6^VgatCre^* animals buried a significantly lower number of marbles than control littermates; remarkably, 44% (8/18) of the *Dlx5/6^VgatCre^* animals displaced less than one marble or no marbles at all (5/18). On the contrary, overexpression of *Dlx5* in *GABAergic^Dlx5/+^* animals resulted in a significant increase in the number of buried or displaced marbles. It should be noted that also in the MBT, the number of buried marbles was linearly correlated to the number of *Dlx5* alleles still present in GABAergic neurons (Figure 4).

− Nest building test

Nest building is an important natural behaviour occurring without the intervention of the experimenter. Whereas *Dlx5/6^VgatCre/+^* and *Dlx5/6^VgatCre^* animals tended to make incomplete and poorly structured nests, even after seven days, overexpression of *Dlx5* in *GABAergic^Dlx5/+^* animals only marginally affected this specific behaviour. Essentially, *GABAergic^Dlx5/+^* animals had a small delay in starting nest construction, but then generated relatively normal nests (Figure 6B). By the end of the test, none of the *Dlx5/6^VgatCre^* animals had built a high-quality nest, whereas all control and *GABAergic^Dlx5/+^* mice had completed nest construction.

## 4. Discussion

Altered function of prefrontal cortical neuronal networks is associated with several psychiatric conditions, including autism and schizophrenia. It has been proposed that changes in the relative activity of excitatory and inhibitory neurons (E/I ratio), which regulates network tuning, can be at the origin of the symptoms observed in these conditions [8,47,48]. PV-positive GABAergic inhibitory interneurons constitute a relatively small part of the global neuronal population, but play a central role in determining the E/I ratio [49]. Several types of cortical PV interneurons can be identified on the basis of their morphological or molecular characteristics [4,5]. PV expressing interneurons are classified into basket, axo-axonic (chandelier), and bistratified cells [50]. These cells are involved in regulating local circuit function and rhythmogenesis and modulate information processing. PV interneurons, are involved in a variety of functions, including local circuit operations, learning and memory, sensory processing, and critical period plasticity. Dysfunctions in PV-interneurons are clearly implicated in autism and schizophrenia [48,51]. Post-mortem studies on patient brains have revealed an important reduction in the density of inhibitory PV-positive neurons both in autism and schizophrenia [52,53,54]. Remarkably, several mouse models of both autism and schizophrenia present a reduction in cortical PV neuronal density reminiscent of what has previously been observed in psychiatric patients (see, for example: ([55,56,57,58,59]). In these models, the reduction in PV neuronal density is due to reduce *Parvalbumin* expression and not due to neuronal cell death [55].

*Dlx5* and *Dlx6* homeobox genes are expressed by developing and mature GABAergic cortical interneurons. Transplantation of immature neurons lacking *Dlx5* or *Dlx5/6* into normal brain has shown a specific reduction in their differentiation in mature PV-interneurons [19]. In the same study, no difference in the density of PV-interneurons was detected in the somatosensory cortex of mice heterozygous for a systemic *Dlx5/6* deletion (*Dlx5/6^+/−^*); this finding is in line with our present observation that PV-interneurons density is normal in the SSC of *Dlx5/6^VgatCre/+^* mice.

The importance of *Dlx5/6* expression in the prefrontal cortex (PFC) has been shown in *Dlx5/6^+/−^* mice, [20] in which the activity of fast-spiking PV interneurons, that generate gamma oscillations, becomes abnormal after adolescence in parallel with the onset of cognitive inflexibility. Although these findings suggest that a reduction in *Dlx5/6* expression could represent a good functional model of schizophrenia [20], the density of PV-interneurons in the prefrontal cortex of *Dlx5/6^+/−^* mice was not measured.

Together these results suggest that *Dlx5/6* expression levels in the PFC could be associated with autistic and/or schizophrenic phenotypes through regulations affecting the PV interneuronal population. In this study, we have targeted the induction of *Dlx5* or deletion of *Dlx5/6* expression specifically to GABAergic neurons to explore the role of these genes on PFC PV neuronal density and behaviour.

Our results reveal a linear correlation between *Dlx5/6* level of expression and PV neuronal density in the prelimbic cortex and in the hippocampus (Figure 4), but not in the somatosensory cortex. After homozygous deletion of *Dlx5/6* in GABAergic neurons in *Dlx5/6^VgatCre^* mice, the density of PV positive neurons in the prelimbic cortex is less than half of that observed in control mice (Figure 2), a proportion similar to what had been observed grafting *Dlx5/6*-null immature neuroblasts [19].

The contrasting results obtained in the frontal and in the somatosensory cortex could indicate that *Dlx5/6* expression is one among other regulators of PV expression, the weight of these factors being different in a primary sensory region such as the somatosensory cortex and in an associative region such as the frontal cortex. Another explanation could be that at least two populations of PV interneurons exist, one sensitive and the other insensitive to *Dlx5/6* expression. In this case, the difference in PV neuron density between regions would mirror differential proportions of these PV neurons subclasses.

In the PFC, *Parvalbumin* levels of expression are variable in adults, and are regulated by several factors, including neuronal activity, serotonin levels and ageing [60], expression variations can even be recorded within a day [61].

In our experiments, the comparison of PV neuron density between mice with *Dlx5* overexpression and *Dlx5/6* invalidation could be indicative of the plasticity of PV neuronal proportion that can be mobilised, or decreased, depending on physiological and environmental conditions.

Together, our results suggest that genetic variations in the DNA regions governing *Dlx5/6* brain expression could contribute to individual differences in cognition [62] and, in certain cases constitute genetic risk factors for psychiatric diseases. The involvement of the *DLX5/6* locus in human psychiatric conditions has been recently shown, integrating genetic and genomic data in a transcriptome-wide association study that provides a comprehensive resource for mechanistic insight and therapeutic development [24]. Molecular pathways or bioactive compounds capable of modulating *Dlx5/6* expression could therefore act on the mind and, reciprocally, existing psychotropic drugs could act on *Dlx5/6* regulations.

## Figures and Tables

**Table 1:** Primers used in this study for RT-qPCR.

***Dlx5***	Fw: 5′ TCT CTA GGA CTGACG CAA ACA 3′
	Rv: 5′ GTT ACA CGC CAT AGG GTC GC 3′
***Pgk1***	Fw: 5′ AACCTCCGCTTTCATGTAGAG 3′
	Rv: 5′ GACATCTCCTAGTTTGGACAGTG 3′
***Hprt***	Fw: 5′ CTCATGGACTGATTATGGACAGGAC 3′
	Rv: 5′ GCAGGTCACCAAAGAACTTATAGCC 3′
***Ppia***	Fw: 5′ CAACCCCACCGTGTTCTTCG 3′
	Rv: 5′ GTGTAAAGTCCCACCCTGGC 3′

## Data Availability

The data underlying this article are available on reasonable request from the corresponding author.
